# A Conceptual Bi-Dimensional Risk Assessment Framework in Bleeding Peptic Ulcers

**DOI:** 10.3390/jcm15114231

**Published:** 2026-05-30

**Authors:** Lodovica Langellotti, Flavio Tirelli, Francesca Mangiola, Valerio Pontecorvi, Rosario Landi, Elena Rodolfino, Roberto Iezzi, Helena Pelanda, Fausto Rosa, Sergio Alfieri

**Affiliations:** 1 UOC Chirurgia Digestiva, Fondazione Policlinico Universitario “Agostino Gemelli” IRCCS, 00168 Rome, Italy; lodovica.langellotti@guest.policlinicogemelli.it (L.L.); sergio.alfieri@policlinicogemelli.it (S.A.); 2 Facoltà di Medicina e Chirurgia, Università Cattolica del Sacro Cuore–Sede di Roma, Largo F. Vito 1, 00168 Rome, Italy; flavio.tirelli@policlinicogemelli.it (F.T.); roberto.iezzi@policlinicogemelli.it (R.I.); helena.pelanda@icatt.it (H.P.); 3 UOC Endoscopia Chirurgica, Fondazione Policlinico Universitario “Agostino Gemelli” IRCCS, 00168 Rome, Italy; francesca.mangiola@policlinicogemelli.it (F.M.); valerio.pontecorvi@policlinicogemelli.it (V.P.); rosario.landi@policlinicogemelli.it (R.L.); 4 Facoltà di Medicina e Chirurgia, Università Cattolica del Sacro Cuore, Centre for Endoscopic Research Therapeutics and Training (CERTT), 00168 Rome, Italy; 5 Department of Diagnostic Imaging and Radiation Oncology, Fondazione Policlinico Universitario “Agostino Gemelli” IRCCS, 00168 Rome, Italy; elena.rodolfino@policlinicogemelli.it; 6 Radiologia Interventistica Avanzata, Dipartimento di Bioimmagini e Radioterapia Oncologica, Fondazione Policlinico Universitario “Agostino Gemelli” IRCCS, 00168 Rome, Italy

**Keywords:** peptic ulcer bleeding, Forrest classification, rebleeding, penetrating ulcer, transcatheter arterial embolization, risk stratification

## Abstract

Peptic ulcer disease remains one of the leading causes of non-variceal upper gastrointestinal bleeding. Despite advances in endoscopic therapy and pharmacological management, recurrent bleeding continues to represent a major cause of morbidity and mortality. Risk stratification is traditionally based on endoscopic stigmata according to the Forrest classification; however, ulcers with similar endoscopic findings may exhibit markedly different clinical outcomes. Increasing evidence suggests that ulcer-related anatomical factors, including size, location, and depth of penetration, may influence the risk of severe or recurrent hemorrhage, particularly in cases involving adjacent arterial structures. In this conceptual, hypothesis-generating review, we propose a conceptual bi-dimensional framework integrating endoscopic and anatomical determinants of bleeding risk. This approach aims to improve patient stratification by identifying a subgroup at “very-high-risk” of recurrent bleeding, in whom standard endoscopic therapy alone may be insufficient. Although this framework is hypothesis-generating and not yet validated, it may provide a conceptual basis for future studies aimed at improving individualized management strategies, including early imaging assessment and consideration of transarterial embolization in selected high-risk patients.

## 1. Introduction

Peptic ulcer (PU) disease remains one of the leading causes of non-variceal upper gastrointestinal bleeding (NVUGIB) and accounts for the majority of acute upper gastrointestinal bleeding episodes worldwide, for approximately 35 to 60% of cases. PU disease affects approximately 4 million individuals worldwide each year, with an estimated global prevalence of over 8 million cases. Although precise global hospitalization data are lacking, complications such as ulcer bleeding account for approximately 19–57 hospitalizations per 100,000 population annually [1,2,3]. Despite the widespread use of antisecretory agents and endoscopic hemostasis techniques, mortality associated with upper gastrointestinal bleeding remains substantial, ranging between 3 and 14.6% [1,2,3]. Rebleeding represents one of the major burdens leading to increased mortality, need for surgery, and prolonged hospitalization [1,2,3]. Clinical risk assessment in bleeding PUs is traditionally based on endoscopic stigmata of recent hemorrhage, most commonly classified according to the Forrest Classification, which is employed for therapeutic decision-making, allowing the identification of lesions that require urgent endoscopic therapy [2,4,5].

However, this system is based on the luminal manifestation of bleeding without taking into account the underlying vascular anatomy of the ulcer. Clinical experience suggests that ulcers with similar endoscopic appearances may exhibit markedly different clinical outcomes [1,2]. These observations suggest that factors beyond the endoscopic bleeding stigmata may contribute to the risk of recurrent hemorrhage. In particular, the anatomical characteristics of the ulcer—specifically its depth and potential penetration into adjacent vascular structures—may play an important role in determining bleeding severity and recurrence [1,2].

Despite advances in endoscopic therapy, recurrent bleeding remains a major clinical challenge, suggesting that current prognostic models may not fully capture all determinants of bleeding severity. Current risk stratification models only partially incorporate ulcer-related anatomical features, which may play a critical role in determining the source and severity of bleeding.

This narrative review proposes a conceptual framework for bi-dimensional risk assessment in bleeding PU, integrating traditional endoscopic risk stratification with anatomical features related to ulcer penetration and vascular involvement. Understanding the interaction between these two dimensions may help explain the heterogeneity of clinical outcomes observed in patients with bleeding ulcers and may support the development of more individualized management strategies.

## 2. Materials and Methods

This study was conducted as a narrative, non-systematic review, aimed at summarizing current evidence on risk stratification in PU bleeding and exploring the potential role of anatomical factors in predicting recurrent hemorrhage.

A literature search was conducted using PubMed/MEDLINE, Scopus, and Google Scholar up to April 2026. The following search terms and their combinations were used: “peptic ulcer bleeding”, “non-variceal upper gastrointestinal bleeding”, “rebleeding”, “risk stratification”, “Forrest classification”, “ulcer penetration”, “deep ulcer”, “pseudoaneurysm”, “transcatheter arterial embolization”. These terms were combined using AND/OR to identify relevant publications.

Given the narrative nature of this review, a formal systematic selection process was not applied. Instead, studies were selected based on their relevance to the topic and their contribution to the conceptual framework.

Priority was given to clinical trials and observational studies, studies investigating predictors of rebleeding, research addressing ulcer size, location, and penetration, studies on transcatheter arterial embolization, international guidelines and consensus statements. Additional relevant articles were identified through manual screening of reference lists of selected publications.

This review was not intended to be a systematic review or meta-analysis. Therefore, no formal quality assessment or quantitative synthesis was performed. The aim was to provide a conceptual synthesis of existing evidence to support the proposed bi-dimensional framework.

## 3. Endoscopic Risk Stratification and Existing Clinical Scores

### 3.1. The Forrest Classification

The Forrest Classification, introduced in 1974, categorizes bleeding PUs according to endoscopic stigmata of recent hemorrhage and remains widely used for risk stratification and therapeutic decision-making [6]. Furthermore, it has proven highly valuable in clinical practice and remains a cornerstone of current management guidelines. By stratifying patients according to the endoscopic appearance of the ulcer base, it allows clinicians to estimate the risk of rebleeding and guide the need for endoscopic therapy and intensive monitoring. Rebleeding risk, mortality, and need for surgery according to the Forrest Classification are summarized in Table 1. It should be noted that the classical rebleeding rates associated with the Forrest classification are largely derived from untreated lesions and may not reflect outcomes in contemporary practice, where endoscopic hemostasis and proton pump inhibitor (PPI) therapy significantly reduce the risk of recurrence.

According to the ESGE guidelines, PUs with active spurting or oozing bleeding (Forrest Ia–Ib) and ulcers with a nonbleeding visible vessel (Forrest IIa) should receive endoscopic hemostatic therapy because of their high risk of persistent bleeding or rebleeding (strong recommendation, high-quality evidence). Endoscopic hemostasis, particularly when combined with PPI therapy, has been shown to significantly reduce recurrent bleeding and need for surgery in these high-risk lesions. In contrast, ulcers with a flat pigmented spot (Forrest IIc) or clean base (Forrest III) carry a low risk of recurrent bleeding, and endoscopic hemostasis is generally not indicated. The role of endoscopic therapy in ulcers with adherent clot (Forrest IIb) remains more controversial. While several studies and meta-analyses suggest a reduction in recurrent bleeding following endoscopic therapy, conflicting evidence persists regarding its impact on surgery and mortality outcomes [2].

Importantly, although the Forrest classification has consistently demonstrated prognostic value in predicting recurrent bleeding, current guidelines also recognize that additional ulcer-related features may influence outcomes and endoscopic treatment failure. These include large ulcer size (>2 cm), posterior duodenal location, large-caliber visible vessels, and the presence of blood within the gastric lumen [2,7,8].

These findings highlight an important limitation, which primarily reflects the luminal appearance of bleeding but may not fully capture the underlying anatomical substrate or the extent of vascular involvement. Within this context, integrating endoscopic stigmata with anatomical ulcer characteristics may provide a more comprehensive conceptual framework for understanding bleeding severity and recurrent hemorrhage risk.

The Forrest classification stratifies ulcers according to the endoscopic appearance of bleeding or stigmata of recent hemorrhage. High-risk lesions include active bleeding (Forrest Ia–Ib) and non-bleeding visible vessels (Forrest IIa), which are associated with a substantial risk of recurrent bleeding if left untreated. Ulcers with low-risk stigmata (Forrest IIc–III) have significantly lower rates of rebleeding and often require only conservative management. These estimates are derived from historical cohorts of untreated PUs and may overestimate the risk of rebleeding in the setting of modern endoscopic and pharmacological therapy.

### 3.2. Risk Scores

Risk assessment represents a crucial step in the management of patients presenting with non-variceal upper gastrointestinal bleeding, as it helps guide decisions regarding the intensity of monitoring, timing of endoscopy, and need for therapeutic intervention. In addition to endoscopic findings, several clinical risk scores have been developed to integrate endoscopic features with clinical and laboratory variables. The Rockall score, for example, incorporates both pre-endoscopic and post-endoscopic parameters, including patient age, presence of shock, comorbidities, endoscopic diagnosis, and stigmata of recent hemorrhage, to estimate the risk of rebleeding and mortality, and to help stratify which patients need endoscopy and intensive care [9]. The complete Rockall score demonstrates a stepwise association with both rebleeding and mortality, with outcomes worsening progressively as the score increases. Increasing scores are associated with progressively higher risks of rebleeding and mortality, with mortality rates rising from 0% in low-risk patients (score 0–1) to over 40% in patients with scores ≥ 8 [9].

Similarly, the Glasgow-Blatchford score (GBS) is a validated pre-endoscopic tool used to stratify the risk of patients presenting with upper gastrointestinal bleeding. This score relies exclusively on clinical and laboratory variables obtained before endoscopy, including hemoglobin level, blood urea nitrogen, systolic blood pressure, heart rate, presentation with melena or syncope, and the presence of hepatic disease or cardiac failure [10]. The GBS ranges from 0 to 23, with higher values associated with increased clinical acuity, mortality risk, and need for therapeutic intervention. Patients with a score of 0 are considered at very low risk and may be suitable for outpatient management, whereas scores above 0 should be interpreted within the broader clinical context rather than as an absolute indication for admission [10].

Moreover, the AIMS65 score has been proposed as a simplified tool for predicting in-hospital mortality in patients with upper gastrointestinal bleeding, developed to predict in-hospital mortality. This score is based on five variables: albumin level, international normalized ratio (INR), altered mental status, systolic blood pressure, and age ≥ 65 years [8]. Although the AIMS65 score performs well in predicting mortality, the Glasgow-Blatchford Score (GBS) has demonstrated greater sensitivity for identifying low-risk bleeding and superior performance in predicting the need for intervention or rebleeding. Therefore, a low AIMS65 score alone should not be used to guide discharge decisions. The score was retrospectively derived from a large cohort and validated in extensive populations; however, its association with endoscopic outcomes, transfusion requirements, and rebleeding risk remains less clearly defined. Importantly, hemodynamic stabilization should always precede formal risk assessment.

**Figure 1:**
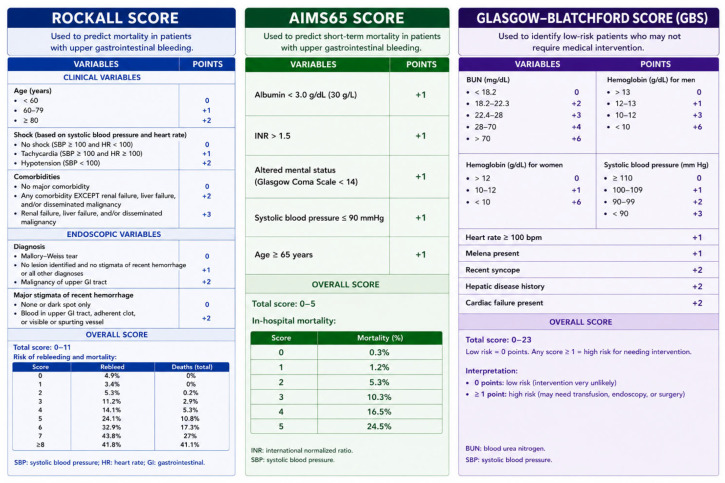
Comparison of the Rockall Score, AIMS65, and Glasgow-Blatchford Score (GBS) for upper gastrointestinal bleeding risk stratification, including variables, scoring criteria, and principal clinical outcomes predicted by each tool.

Although each scoring system was developed with different clinical objectives, the Rockall Score, AIMS65, and Glasgow-Blatchford Score (GBS) collectively represent the cornerstone of contemporary risk stratification in upper gastrointestinal bleeding. Figure 1 summarizes their principal variables, score ranges, and clinical implications.

## 4. Beyond Endoscopic Stigmata: The Anatomy of Bleeding Ulcers

Although the Forrest classification remains the cornerstone of endoscopic assessment of bleeding severity, increasing evidence suggests that ulcer-related anatomical characteristics may independently influence the risk of recurrent hemorrhage and treatment failure.

Increasing evidence suggests that ulcer-related anatomical factors, including ulcer size, location, and depth of penetration, may significantly influence the likelihood of severe or recurrent hemorrhage [2,11,12]. These anatomical factors are mainly evaluated endoscopically, enabling direct assessment of ulcer size, location, and depth of penetration; at the same time radiologic imaging may provide complementary information in selected cases, particularly for assessing transmural involvement or associated complications.

Several studies have identified large ulcer size as a major predictor of adverse outcomes [11,12,13,14,15]. In a multicenter study including 1264 patients with PU bleeding, Camus et al. [13] demonstrated that ulcers larger than 2 cm were independently associated with increased 30-day rebleeding, need for surgery, and mortality. Similarly, Elmunzer et al. [12], in a systematic review of predictors of recurrent hemorrhage after endoscopic hemostatic therapy, identified ulcer size, posterior duodenal location, hemodynamic instability, and active bleeding at presentation as consistent predictors of recurrent bleeding.

**Figure 2:**
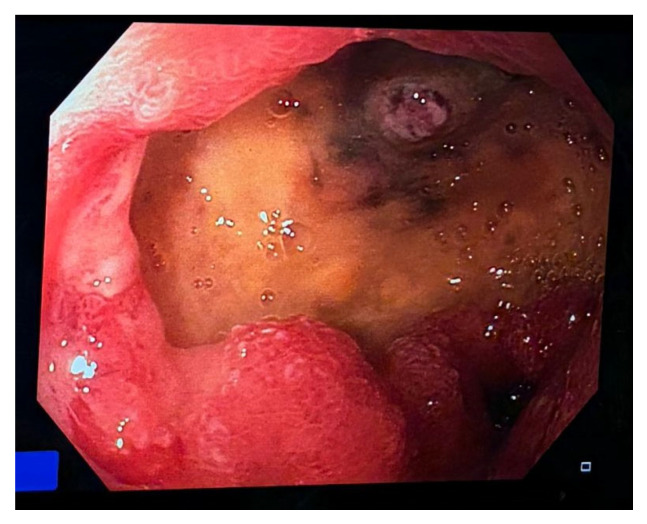
Endoscopic view of a deep ulcer located in the second portion of the duodenum, showing full-thickness perforation with direct visualization of the underlying pancreatic parenchyma. Images are anonymized and used for illustrative purposes only.

Anatomical location also appears to play a critical role. Ulcers located on the posterior wall of the duodenal bulb are particularly prone to severe hemorrhage because of their close proximity to the gastroduodenal artery [16]. In these patients, endoscopic hemostasis may achieve temporary luminal control while failing to address deeper arterial involvement or pseudoaneurysm formation. In particular, some PUs extend beyond the mucosal and submucosal layers and penetrate deeper tissues, a process commonly referred to as a penetrating ulcer. For clarity, “deep ulcers” are defined as lesions extending beyond the submucosa, “penetrating ulcers” as those extending into adjacent structures beyond the muscularis propria, and “full-thickness ulcers” as lesions involving the entire wall thickness with transmural extension. This phenomenon occurs most frequently in the posterior wall of the duodenal bulb, which accounts for approximately 60–70% of duodenal ulcers. Although precise estimates vary, a subset of ulcers demonstrate deep or penetrating behavior, which has been associated with increased risk of arterial erosion and severe hemorrhage where the duodenum lies near major vascular structures, including branches of the gastroduodenal artery. In this anatomical setting, progressive ulcer erosion may involve larger arterial vessels supplying the duodenum rather than superficial submucosal vessels, predisposing to more severe hemorrhage and increasing the risk of recurrent bleeding even after apparently successful endoscopic hemostasis [2,11,12] (Figure 2).

Importantly, predictors of failure of repeat endoscopic therapy identified in the randomized trial by Lau et al. [11] included hypotension during recurrent bleeding and ulcer size >2 cm, suggesting that anatomical and hemodynamic factors may substantially influence outcomes beyond endoscopic stigmata alone. These findings are consistent with current ESGE guideline statements acknowledging that ulcer size and anatomical location may influence treatment outcomes independently of Forrest classification.

**Figure 3:**
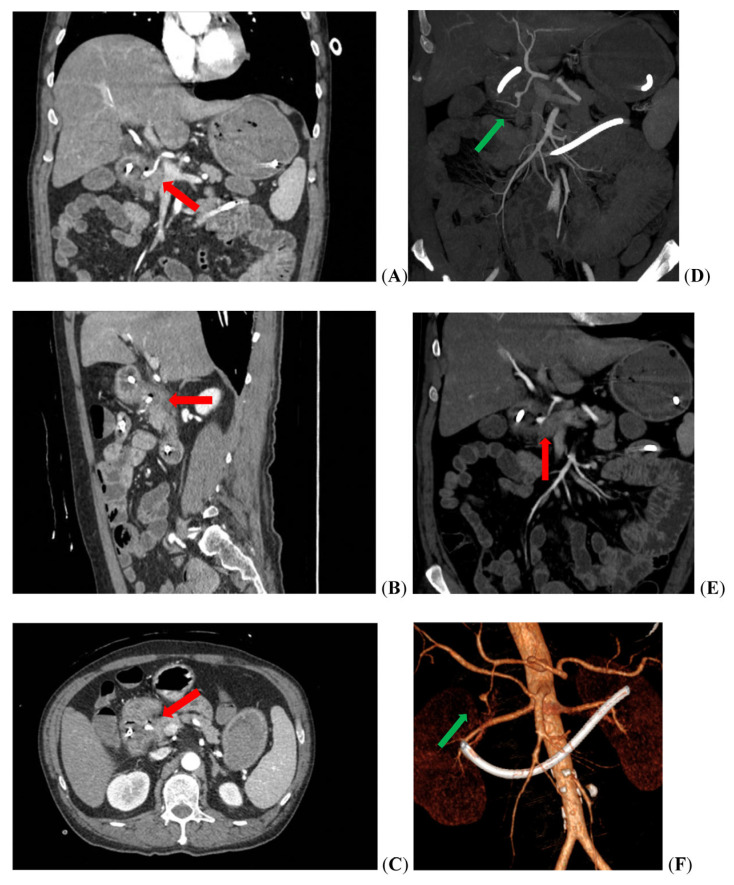
Contrast-enhanced CT with multiplanar reconstructions demonstrating a penetrating duodenal ulcer involving the posterior wall of the second portion of the duodenum. Coronal (**A**), sagittal (**B**), axial (**C**), MIP reconstruction of the CT-angiography (**D**), MPR reconstruction of the CT-angiography (**E**), and three-dimensional volume-rendered CT reconstruction (**F**) images show focal discontinuity of the posterior duodenal wall with an adjacent peri-duodenal fluid collection. The duodenal bulb region showed inflammatory changes including a mural thickening and hyper enhancing mucosa. Images depict a focal luminal outpouching with disruption in the normal enhancement of posterior duodenal bulb wall corresponding to a deep ulcer crater filled by fluid and air (red arrow) with stranding of the adjacent fat tissue. Within the collection, a well-defined contrast-enhancing focus consistent with a pseudoaneurysm of the gastroduodenal artery is identified (green arrow). These findings are compatible with deep ulcer penetration and arterial involvement. In these cases, bleeding may arise from major arterial branches rather than from superficial submucosal vessels. This anatomical relationship provides a pathophysiological explanation for severe or recurrent hemorrhage and highlights the potential limitations of endoscopic therapy alone in deeply penetrating ulcers. Images are anonymized and used for illustrative purposes only.

Collectively, these findings support the hypothesis that the luminal appearance of an ulcer may not fully reflect the severity of the underlying vascular lesion. Within this context, integrating endoscopic findings with anatomical ulcer characteristics may provide a more comprehensive conceptual framework for understanding recurrent or catastrophic bleeding in selected high-risk patients. Furthermore, chronic inflammation surrounding penetrating ulcers may weaken the arterial wall and occasionally promote the formation of visceral arterial pseudoaneurysms, which can subsequently rupture into the gastrointestinal lumen and cause delayed or recurrent bleeding episodes [17,18]. In such cases, bleeding may be difficult to control with endoscopic therapy alone and may require interventional radiology or surgical management. (Figure 3).

In rare instances, deeply penetrating ulcers may also involve adjacent biliary structures, leading to complications such as haemobilia or biliary fistulas, further illustrating how the anatomical behavior of the ulcer can influence the clinical presentation and course of gastrointestinal bleeding [19,20].

From a clinical perspective, anatomical risk may be inferred through surrogate markers, including large ulcer size (≥2 cm), posterior duodenal location, evidence of deep penetration on imaging, or proximity to major arterial structures such as the gastroduodenal artery. These features may help identify ulcers in which bleeding originates from larger arterial vessels rather than superficial submucosal branches. Taken together, these observations suggest that ulcer location and depth of penetration represent an additional anatomical dimension of bleeding risk, complementing traditional endoscopic classifications. Integrating these anatomical considerations with endoscopic findings may therefore provide a more comprehensive assessment of hemorrhage severity and recurrence risk in patients with PU bleeding.

## 5. A Conceptual Bi-Dimensional Risk Assessment Framework

The observations discussed above suggest that risk stratification in bleeding PUs may benefit from integrating two complementary dimensions: the endoscopic dimension, represented by the Forrest classification, and the anatomical dimension, reflecting ulcer penetration and potential vascular involvement, both primarily evaluated endoscopically at the time of index endoscopy, while radiologic imaging may offer additional insights in selected cases, particularly for assessing deeper penetration or vascular involvement.

### 5.1. Endoscopic Dimension

This dimension reflects the luminal manifestation of bleeding and is based on the Forrest classification. Lesions are categorized as high-risk endoscopic stigmata (Ia, Ib, IIa, and IIb) and low-risk endoscopic stigmata (IIc and III).

### 5.2. Anatomical Dimension

This dimension reflects ulcer depth and the likelihood of involvement of adjacent vascular structures. In the absence of standardized definitions, anatomical risk is defined using pragmatic surrogate markers derived from available evidence and clinical practice.

Within the conceptual framework proposed in this work, ulcers may be pragmatically categorized according to anatomical features potentially associated with deeper vascular involvement and increased bleeding severity. Low anatomical risk refers to superficial ulcers without features suggestive of deep penetration or vascular involvement, whereas high anatomical risk includes ulcers presenting one or more of the following characteristics: ulcer size ≥ 2 cm, posterior duodenal location, suspected deep/transmural penetration, or proximity to major arterial structures. These proposed criteria are intended as exploratory surrogate markers derived from available evidence and clinical reasoning, rather than as a validated classification system [11,12,13,14,15,16].

These criteria are intended to reflect an increased likelihood of arterial erosion and clinically significant bleeding [11,12,13,14,15,16,17,18].

Within this proposed framework, the risk of recurrent bleeding may be influenced by the interaction between endoscopic severity and the anatomical depth of ulcer penetration. Ulcers characterized by both high-risk endoscopic stigmata and significant anatomical penetration—such as posterior duodenal ulcers eroding into major arterial branches—may represent the subgroup with the highest risk of recurrent hemorrhage.

Conversely, ulcers with high-risk endoscopic stigmata but limited anatomical penetration may respond well to standard endoscopic therapy. Similarly, ulcers with minimal endoscopic bleeding activity but significant penetration may occasionally present with delayed or atypical bleeding patterns.

**Figure 4:**
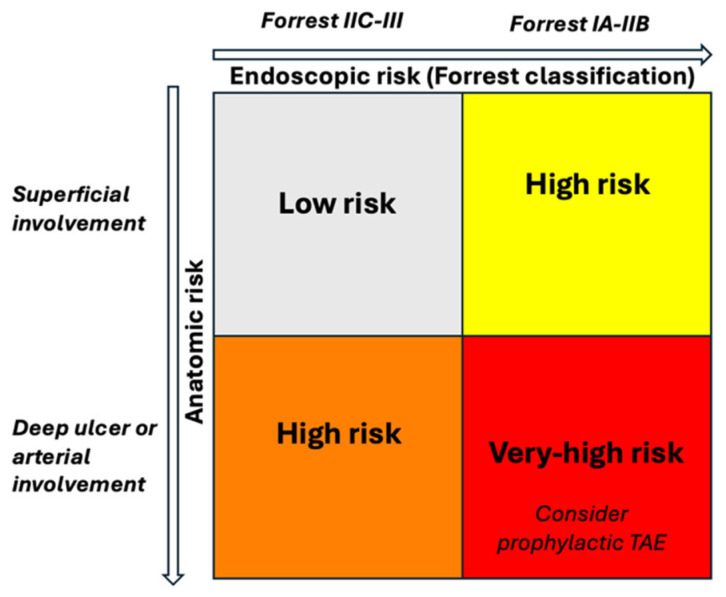
Bi-dimensional framework integrating endoscopic and anatomical risk in bleeding PU. Traditional risk stratification is primarily based on endoscopic stigmata according to the Forrest classification, distinguishing low-risk (Forrest IIc–III) from high-risk lesions (Forrest Ia–IIb). The proposed framework incorporates an additional anatomical dimension reflecting ulcer depth and the likelihood of involvement of adjacent arterial structures. Ulcers with superficial involvement are classified as low anatomical risk, whereas those with deep penetration and potential arterial involvement are classified as high anatomical risk. The combination of these two dimensions identifies a subgroup of patients at very-high-risk of recurrent bleeding, characterized by both high-risk endoscopic stigmata and deep anatomical involvement, who may benefit from more aggressive therapeutic strategies, including consideration of prophylactic transcatheter arterial embolization.

In this context, the proposed bi-dimensional framework may provide a conceptual model for understanding the interaction between endoscopic and anatomical determinants of bleeding risk, with the aim of improving patient stratification and identifying subgroups who may benefit from tailored therapeutic strategies. This conceptual model is illustrated in Figure 4.

The proposed model should be regarded as an exploratory framework integrating existing evidence and requiring prospective validation before clinical implementation. In this framework, anatomical risk should be interpreted using surrogate markers such as ulcer size (>2 cm), posterior duodenal location, CT evidence of penetration, or proximity to major arterial structures.

## 6. Clinical Implications: The Role of Prophylactic Embolization

Current international guidelines for PU bleeding primarily rely on endoscopic risk stratification based on the Forrest classification, which guides decisions regarding endoscopic hemostasis, proton pump inhibitor therapy, monitoring intensity, and escalation of care [2,4,6]. In patients with recurrent bleeding after initially successful endoscopic treatment, both European and American guidelines recommend repeat endoscopy with additional hemostatic therapy as the preferred first-line approach.

The 2015 European Society of Gastrointestinal Endoscopy (ESGE) guidelines recommended to repeat endoscopy in cases of recurrent bleeding and suggested transcatheter angiographic embolization (TAE) or surgery only after failure of a second endoscopic attempt (strong recommendation, high-quality evidence) [2]. The updated 2021 ESGE guidelines further refined this strategy by recommending consideration of cap-mounted clips during repeat endoscopic therapy, while positioning TAE as the preferred rescue option after failed endoscopic hemostasis, with surgery reserved for cases in which embolization is unavailable or unsuccessful (strong recommendation, moderate-quality evidence) [21].

Similarly, the 2021 American College of Gastroenterology (ACG) guidelines suggest repeat endoscopy and endoscopic therapy rather than surgery or TAE in patients with recurrent ulcer bleeding after initial endoscopic hemostasis (conditional recommendation) [22]. This recommendation is largely based on the randomized trial by Lau et al., in which repeat endoscopic therapy controlled recurrent bleeding in approximately three-quarters of patients and was associated with fewer complications than surgery. Notably, the same study identified hypotension during rebleeding episodes and ulcer size > 2 cm as predictors of failure of repeat endoscopic therapy [23].

Despite these advances, current guideline-based risk stratification remains predominantly centered on endoscopic stigmata and may not fully account for ulcer-related anatomical factors contributing to bleeding severity and recurrence. In clinical practice, ulcers with similar endoscopic appearances may arise from substantially different anatomical substrates, ranging from superficial mucosal vessel exposure to erosion of major arterial branches, particularly in anatomically vulnerable regions such as the posterior wall of the duodenal bulb adjacent to the gastroduodenal artery.

Within this context, the proposed bi-dimensional framework is intended to complement existing guideline-based pathways by integrating endoscopic findings with anatomical features associated with deep penetration and vascular involvement. This conceptual approach may help identify a subgroup of patients at “very-high-risk” of recurrent bleeding, characterized by the coexistence of high-risk endoscopic stigmata and unfavorable anatomical features such as large ulcer size, posterior duodenal location, or evidence of transmural penetration.

Importantly, prophylactic transcatheter arterial embolization (PTAE) following successful endoscopic hemostasis is not routinely recommended in current guidelines and remains an investigational strategy in carefully selected high-risk patients. However, the identification of patients with combined high-risk endoscopic and anatomical features may provide a rationale for future studies evaluating intensified or alternative therapeutic approaches, including earlier radiologic assessment or prophylactic embolization [21,22,23,24,25,26].

Ultimately, management of these complex patients should rely on multidisciplinary collaboration involving gastroenterologists, endoscopists, interventional radiologists, and surgeons [21,22]. Importantly, the proposed framework should be interpreted as hypothesis-generating and not as a validated tool for direct clinical decision-making. An illustrative clinical vignette exemplifying the application of the proposed bi-dimensional framework is provided in the Supplementary Material (Figure S1).

## 7. Conclusions and Future Perspective

The bi-dimensional framework proposed in this review may therefore help identify a potential subgroup of patients at “very-high-risk” of rebleeding. These patients not only exhibit high-risk endoscopic stigmata but also harbor unfavorable anatomical features (e.g., duodenal ulcers with full-thickness penetration), which may predispose them to severe or recurrent bleeding and potentially justify prophylactic transarterial embolization. This subgroup may represent an underrecognized population in current management algorithms, where risk assessment is largely based on endoscopic stigmata while anatomical features of the ulcer are less systematically considered. However, this framework requires prospective validation before any clinical application can be considered. Future prospective studies are needed to validate this proposed risk category and to determine whether integrating ulcer-related anatomical factors into existing prognostic models may improve patient selection for preventive strategies such as prophylactic transarterial embolization. Incorporating both endoscopic stigmata of active or recent bleeding and ulcer-related anatomical features into a unified risk framework may ultimately enable a more refined identification of patients at the highest risk of adverse outcomes and support a more tailored therapeutic approach.

## Figures and Tables

**Table 1:** Forrest classification and associated risk of rebleeding, need for surgery, and mortality in PU bleeding.

Forrest Class	Endoscopic Finding	Risk of Rebleeding (Within 72 h)	Need for Surgery	Mortality
Ia	Active spurting bleeding	90–100%	35%	11%
Ib	Active oozing bleeding	~80%	35%	11%
IIa	Non-bleeding visible vessel	40–60%	34%	11%
IIb	Adherent clot	20–25%	10%	7%
IIc	Flat pigmented spot (hematin on ulcer base)	<13%	6%	3%
III	Clean ulcer base	<5%	0.5%	0–2%

## Data Availability

No new data were created or analyzed in this study.

## Supplementary Materials

The following supporting information can be downloaded at: https://www.mdpi.com/article/10.3390/jcm15114231/s1, Figure S1: Illustrative clinical vignette applying the bi-dimensional framework.
