# Supplementary material for: A Conceptual Bi-Dimensional Risk Assessment Framework in Bleeding Peptic Ulcers
Source: J Clin Med. 2026 May 30;15(11):4231. doi: 10.3390/jcm15114231 (PMC13257769; doi:10.3390/jcm15114231)
Supplement: Supplementary file 1 [file jcm-15-04231-s001.zip › jcm-4281496-supplementary.pdf]

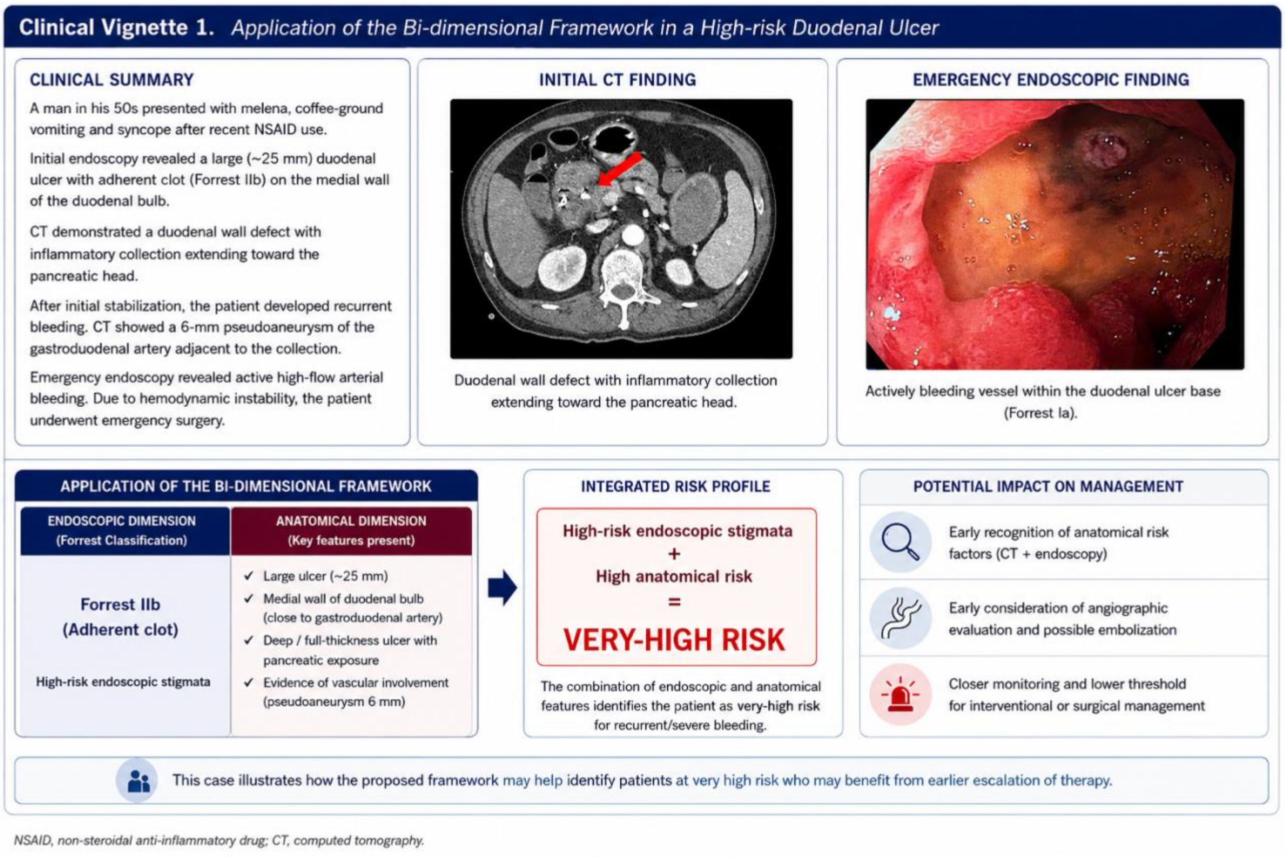

Supplementary Material Figure S1. Illustrative clinical vignette applying the bi-dimensional framework.

Representative endoscopic and radiologic findings from an illustrative clinical scenario are shown.
Upper endoscopy demonstrating a large duodenal ulcer with an adherent clot, consistent with Forrest IIb classification.
Contrast-enhanced CT showing a focal vascular lesion (arrow) adjacent to the duodenal wall, consistent with a pseudoaneurysm of the gastroduodenal artery in the setting of deep ulcer penetration.
The coexistence of high-risk endoscopic stigmata and unfavorable anatomical features (large size, posterior location, deep penetration, and vascular involvement) is consistent with a “very-high-risk” profile within the proposed bi-dimensional framework.
This example illustrates how integration of endoscopic and anatomical findings may help conceptualize patients at increased risk of severe or recurrent bleeding.
These images are provided for illustrative purposes only and do not represent a specific identifiable patient.
